# Complete genome sequence of *Oenococcus oeni* strain K19-3 isolated from grape must

**DOI:** 10.1128/mra.00988-23

**Published:** 2023-12-13

**Authors:** Andrey V. Mardanov, Alexey V. Beletsky, Egor A. Vasyagin, Tatiana N. Tanashchuk, Maxim Yu. Shalamitskiy, Nikolai V. Ravin

**Affiliations:** 1 Institute of Bioengineering, Research Center of Biotechnology of the Russian Academy of Sciences, Moscow, Russia; 2 Research Institute of Viticulture and Winemaking “Magarach” of the Russian Academy of Sciences, Yalta, Russia; Portland State University, Portland, Oregon, USA

**Keywords:** *Oenococcus oeni*, genome, malolactic fermentation

## Abstract

The lactic acid bacteria *Oenococcus oeni* spp. are of significant interest in winemaking due to their ability to carry out malolactic fermentation, thereby improving the organoleptic properties of wine. Here we report the complete circular genome sequence of the *Oenococcus oeni* strain К19-3, isolated from red grape must at Crimean wineries.

## ANNOUNCEMENT

*Oenococcus oeni*, the lactic acid bacteria, plays a vital role in winemaking by providing stability, reducing acidity, and influencing the sensory characteristics of various wine styles through the process of malolactic fermentation (MLF) (1). However, it is sensitive to harsh conditions during wine fermentation, such as high ethanol concentration, low pH, and sulfur dioxide.

The Magarach Collection of Microorganisms for Winemaking contains a large number of yeast and bacterial wine strains isolated from natural sources and obtained from other collections (2). The *O. oeni* strain K19-3 was isolated in 2012 from fermented red grape must at the “Zolotaya Balka” winery, Balaklava District, Sevastopol, Crimea. The isolate was grown in De Man, Rogosa, and Sharpe medium (3) supplemented with 10 (vol/vol) ethanol and 5-g/L malic acid at pH 3.0 and underwent MLF.

Here we report the complete genome of *O. oeni* K19-3 obtained using two high-throughput sequencing platforms, Illumina MiSeq (Illumina Inc., San Diego, CA, USA) and MinION (Oxford Nanopore, Oxford, UK). The strain was grown in MRS medium at 28C for two days. Total DNA was isolated using the DNeasy PowerSoil Kit (Qiagen, Hilden, Germany). The sequencing of NEBNext Ultra II DNA Library (NEB, Ipswich, USA) generated 627,491 read pairs (2 × 300 nt) with a total length of 377.7 Mb. Overlapping reads were merged using FLASH v.1.2.11 (4), low-quality sequences were trimmed with sickle v.1.33 (https://github.com/najoshi/sickle). Additionally, the *O. oeni* genome library was generated using Ligation Sequencing Kit LSK-109 and sequenced in a FLO-MIN111 flow cell using a MinION device. A total of 31,515 reads with a total length of 63.9 Mb were obtained. The N50 value of reads was 4,680 bp; the average read length was 2,028 bp; and the maximum read length was 101,399 bp. Basecalling was done using guppy v.5.0.7 (5).

Illumina and Nanopore sequences were assembled using HybridSPAdes v.3.15.4 (6) in isolate mode; complete genome sequence was circularized manually by visualizing HybridSPAdes assembly graph in Bandage v.0.8.1. The assembled genome was 1,862,087 bp in size with GC content of 37.83%. Genes were predicted using National Center for Biotechnology Information Prokaryotic Genome Annotation Pipeline v.2023–05-17.build6771 (7). All tools were run with default parameters unless otherwise specified. A total of 1,813 protein-coding genes, 46 tRNA genes, 6 rRNA genes, and 43 pseudogenes were predicted in the genome.

Taxonomic assignment based on complete genomes using the GTDB-Tk v.2.3.0 (reference data v.r214) (8) showed that the closest genome belonged to *O. oeni* DSM 20252 (GCF_000372485.1) with an average nucleotide identity of 99.03%. The data reported in this work will be useful for further research in the field of comparative genomics of *Oenococcus*.

## Data Availability

The whole-genome nucleotide sequence of *Oenococcus oeni* K19-3 3 has been deposited in DDBJ/ENA/GenBank under the accession number CP136429, and raw reads have been deposited in the National Center for Biotechnology Information Sequence Read Archive under the accession numbers SRX21820546 and SRX21820547. The version described in this paper is the first version.
